# Bacterial ribonuclease binase exerts an intra-cellular anti-viral mode of action targeting viral RNAs in influenza a virus-infected MDCK-II cells

**DOI:** 10.1186/s12985-017-0915-1

**Published:** 2018-01-05

**Authors:** Raihan Shah Mahmud, Ahmed Mostafa, Christin Müller, Pumaree Kanrai, Vera Ulyanova, Yulia Sokurenko, Julia Dzieciolowski, Irina Kuznetsova, Olga Ilinskaya, Stephan Pleschka

**Affiliations:** 1 Institute of Fundamental Medicine and Biology, Kazan Federal University, Kremlyovskaya Street 18, 420008 Kazan, Russia; 2 Institute of Medical Virology, Justus Liebig University, Schubertstrasse 81, 35392 Giessen, Germany; 3 Center of Scientific Excellence for Influenza Viruses, National Research Center (NRC), El-Buhouth Street 87, 12311 Dokki, Cairo, Egypt; 4 Present address: Department I - Cardiac Development and Remodelling, Max Planck Institute for Heart and Lung Research, Ludwigstrasse 43, 61231 Bad Nauheim, Germany; 5 Present address: Department of Biochemistry and Molecular Biology, Institute of Nutritional Science, Justus Liebig University, Heinrich-Buff-Ring 26-32, 35392 Giessen, Germany

**Keywords:** Ribonuclease, RNase, Binase, Influenza virus, Anti-viral activity

## Abstract

**Background:**

Influenza is a severe contagious disease especially in children, elderly and immunocompromised patients. Beside vaccination, the discovery of new anti-viral agents represents an important strategy to encounter seasonal and pandemic influenza A virus (IAV) strains. The bacterial extra-cellular ribonuclease binase is a well-studied RNase from *Bacillus pumilus*. Treatment with binase was shown to improve survival of laboratory animals infected with different RNA viruses. Although binase reduced IAV titer in vitro and in vivo, the mode of action (MOA) of binase against IAV at the molecular level has yet not been studied in depth and remains elusive.

**Methods:**

To analyze whether binase impairs virus replication by direct interaction with the viral particle we applied a hemagglutination inhibition assay and monitored the integrity of the viral RNA within the virus particle by RT-PCR. Furthermore, we used Western blot and confocal microscopy analysis to study whether binase can internalize into MDCK-II cells. By primer extension we examined the effect of binase on the integrity of viral RNAs within the cells and using a mini-genome system we explored the effect of binase on the viral expression.

**Results:**

We show that (i) binase does not to attack IAV particle-protected viral RNA, (ii) internalized binase could be detected within the cytosol of MDCK-II cells and that (iii) binase impairs IAV replication by specifically degrading viral RNA species within the infected MDCK-II cells without obvious effect on cellular mRNAs.

**Conclusion:**

Our data provide novel evidence suggesting that binase is a potential anti-viral agent with specific intra-cellular MOA.

**Electronic supplementary material:**

The online version of this article (10.1186/s12985-017-0915-1) contains supplementary material, which is available to authorized users.

## Background

Influenza A virus (IAV) is an RNA virus, which poses a great health risk causing seasonal epidemics and periodically worldwide pandemics. Despite their seasonal character, influenza epidemics are unpredictable and have been recognized as a major cause of morbidity and increased mortality [1]. Influenza virus infection leads to a disease that results in 0.25–0.5 million deaths annually worldwide [2]. Currently, two classes of anti-virals are available for the treatment of seasonal human influenza: neuraminidase inhibitors (oseltamivir, zanamivir, peramivir) and M2-channel blockers (rimantadine and amantadine) acting on viral spread and entry, respectively [3]. Nevertheless, vaccination is still the most effective preventive measure, regardless to the constant changes of the viral antigenic epitopes (antigen drift), demanding the annual renewal of the selected vaccine strains [4, 5]. Furthermore, based on the segmented nature of IAV genome, co-infections can lead to reassortants with completely new antigenic characteristics (antigen shift) that can cause pandemic outbreaks [6, 7]. Thus, the efficacy of currently approved control strategies is limited because IAVs frequently escape the immune response via antigen drift and shift [8]. Therefore, an alternative therapeutic strategy that is effective irrespective of viral subtype by directly attacking the genetic material of the virus would be beneficial.

Along this line, ribonucleases (RNases) might represent an attractive tool [9, 10]. It was previously shown that certain amphibian RNases possess anti-viral activities [11, 12]. Onconase from *Rana pipiens* selectively destroyed the RNA of type-I human immunodeficiency virus (HIV-I) without degrading host RNA molecules [11] and RNase from *Rana catesbeiana* inhibited the replication of Japanese encephalitis virus [12]. Recently, we have shown that binase, an extra-cellular RNase of the gram-positive bacteria *Bacillus pumilus,* reduced the titer of an 2009 pandemic IAV strain (H1N1pdm09) in A549 cells at non-toxic concentrations [13].

Binase is a well-characterized small (12.2 kDa) RNase [9, 14]. Owing to its bacterial origin, binase evades mammalian RNase inhibitors and retains its catalytic activity inside eukaryotic organisms [15]. Binase was previously shown to possess in vitro anti-viral properties against foot-and-mouth disease virus, IAV, reo- and corona- viruses [13, 16–18]. In vivo, binase protected laboratory animals from rabies when it was injected into the site of viral administration and was also efficient against IAV and influenza B virus (IBV) in infected mice [19, 20].

Nevertheless, mechanisms underlying the anti-viral effects of binase remain unclear. We had previously shown that pre-treatment of IAV with binase before infection of A549 and MDCK-II cells resulted in an anti-viral effect that was dependent on the amount of binase [13, 21, 22]. Furthermore, we reported that binase degrades vRNA, which is not protected by the virion/RNP complex and that binase added to cells (HEK293, A549) reduces of a GFP reporter gene expression by a transient IAV mini-genome system [21, 22]. Moreover, we showed that binase is able to penetrate into A549 cells [23] and that treatment of IAV-infected A549 cells led to titer reduction and reduced the viral NP mRNA amount 12 h post-infection (h p.i.) without affecting mRNA levels of cellular housekeeping proteins [22].

Since the anti-viral activity of binase against IAV is evident, it was necessary to investigate whether (i) an extra-cellular effect of binase on the virion and/or (ii) the binding of the virion to the cell surface receptors contributes to the virus titer reduction, and was not investigated what type of viral RNAs are affected by binase within the cell and how this is reflected on the protein level. Also, it was not previously elucidated whether the anti-viral activity of binase coincides with its internalization in other cells (MDCK-II, optimal for IAV propagation).

Hence, we sought to elucidate in more detail the extra- and intra-cellular effect of binase on viral gene expression and protein production in order to establish a more robust basis of its anti-viral mode of action.

## Methods

### Cells and viruses

MDCK-II (Madin-Darby canine kidney epithelial cells) and 293 T (Human embryonic kidney (HEK) constitutively expressing the SV40 large T antigen) (ATCC, USA) were cultured in Dulbecco’s modified Eagle medium (DMEM) (Gibco, USA) supplemented with 10% fetal calf serum (FCS, PAA, Austria), 100 U/ml penicillin and 0.1 mg/ml streptomycin (P/S) (Gibco, USA) and incubated at 37 °C in a 5% CO_2_ atmosphere. Influenza virus A/Hamburg/04/09 (H1N1pdm09) and A/Victoria/3/1975(H3N2-Vict) were propagated in MDCK-II cells at 37 °C and 5% CO_2_. The virus was titrated using focus assay [24, 25] and stored at −80 °C.

### Plasmids and primers

For the in vitro reconstitution of the biological active viral ribonucleoprotein (vRNP) complex, four plasmids encoding the viral PB1, PB2, PA and NP proteins of H1N1pdm09 (pHW-PB1-Hamburg, -PB2-Hamburg, -PA-Hamburg, -NP-Hamburg) together with the plasmid pPol1-CAT-RT generating a vRNA-like Pol1-transcript encoding the chloramphenicol acetyltransferase (CAT) protein, were used. The CAT reporter protein, in negative-sense, is flanked by the 3′- and 5′-noncoding region of the NS-segment of influenza A/WSN/33 virus placed between a truncated human RNA polymerase I promoter (Pol1) and the hepatitis delta virus ribozyme. The expressed subunits of the viral polymerases and nucleoprotein replicate and transcribe the IV-like RNA expressed by pPol1-CAT-RT into mRNA [26].

RT-qPCR primer pairs (ATPase_F: 5′- TGC TCT CTC CTT GGA ACC TGT G -3′; ATPase_R: 5′- GCT CTC CTA CTG ACT GCC TTG TC -3′ and TUB_F: 5′- GGA CTT CAG GGC TTC CTG GTA TTC-3′; TUB_R: 5′- CTT CTT GCC GTA GTC AAC CGA GAG -3′) were used to amplify the cellular housekeeping *ATP6V0E1* ATPase (encoding the enzyme V-type proton ATPase subunit e1) and *TUBA4A* (encoding the cellular structural protein named Tubulin alpha-4A) genes, respectively. The primer pairs were used to identify the binase effect on MDCK-II cellular mRNA after 12 h incubation of MDCK-II cells with- and without binase using real-time RT-PCR. Using a LightCycler 480 (Roche Diagnostics, Switzerland) system, the samples were subjected to the following thermal cycling conditions: 95 °C for 10 min; 40 cycles of 95 °C for 15 s; 60 °C for 30 s; and 30 °C for 30 s.

### Isolation of binase

Binase (secreted guanyl-preferring ribonuclease of 109 amino acid residues in the single chain; EC 3.1.27.3) was isolated from *Bacillus pumilus 7p* culture fluid as described before [18]. Briefly, bacteria were grown until the early stationary phase in a phosphate-deficient medium at 37 °C with agitation at 200 rpm in a Multitron shaking incubator (INFORS HT, Switzerland). The cells were pelleted by centrifugation at 6000 g for 30 min at 4 °C. The supernatant was diluted 1:5 with sterile deionized water after acidification by glacial acetic acid to pH 5.0 and applied on the column packed with DE-32 DEAE cellulose (Whatman, United Kingdom) equilibrated with 10 mM Na acetate buffer (pH 5.0). The eluate was loaded on phosphocellulose P11 (Whatman, United Kingdom) equilibrated with 10 mM Na phosphate buffer (pH 5.0). The phosphocellulose was washed by 10 mM Na phosphate buffer (pH 5.0) until the A_280_ dropped below 0.05. Then, the column was equilibrated with the 20 mM Na phosphate buffer (pH 7.0). The protein was eluted using 200 mM Na phosphate buffer (pH 7.0). Further purification of binase was performed using UNOS6 column equilibrated with 10 mM Na phosphate buffer (pH 7.0) and Biologic DuoFlow chromatography system (Bio-Rad, USA). Protein was eluted using a linear gradient of 0–0.3 M NaCl with the flow rate of 2 mL/min. Protein concentration and RNase activity were determined in the peak fractions. RNase-containing fractions were concentrated and desalted using Amicon Ultra-4 centrifugal filter units (Merck, USA). Homogeneity and authenticity of the purified enzyme were confirmed by PAAG electrophoresis, Western blotting (ECL) and MALDI TOF/TOF mass spectrometry (UltrafleXtreme, Bruker Corporation, Germany) [27]. Protein samples were lyophilized as described before [22].

### RNase activity assay

Ribonuclease activity of binase was measured by the hydrolysis products of high-molecular weight yeast RNA. Reaction mixture containing 0.5 mg/ml of RNA in 0.1 M Tris-HCl buffer, pH 8.5 and solution of binase was incubated at 37 °C for 15 min. The enzymatic reaction was stopped by addition of 6.8% cold perchloric acid on ice. Non-degraded RNA was pelleted by centrifugation at 12000 g for 10 min and the supernatant was used for the measurement of absorption at 260 nm. One unit of ribonuclease activity corresponded to the quantity of the enzyme that increased the absorption at 260 nm by 1 optical unit as calculated for 1 ml of reaction mixture and for 1 h of incubation considering the dilution of the enzyme [13, 18].

### Cytotoxicity concentration 50% (CC_50_)

The cytotoxicity of binase for MDCK-II cells was determined via MTT assay [28]. Briefly, cells incubated with binase for 24 h were washed and incubated for 90 min in 200 μl of MTT-mix (growth DMEM medium containing 175 μg/ml MTT = 1-(4,5-dimethylthiazol-2-yl)-3,5-diphenylformazan; Sigma) and subsequently fixed with 3.7% PFA (paraformaldehyde) for 30 min at room temperature (RT). The cells were dried and the tetrazolium crystals were dissolved by adding 200 μl of isopropanol to each well. The plates were shaken for 10 min and analyzed photometrically at 490 nm excitation in an ELISA reader EL808 (BioTek, USA). The percentage of cell viability after binase exposure was calculated as follows: Percentage viability = 100/(MTT value of untreated sample x MTT value of inhibitor treated sample) using GraphPad Prism 5.01 Software (GraphPad Software, Inc., USA) by plotting the percent of viable cells as a function of the binase concentration.

### Effective concentration 50% (EC_50_)

The effective concentration (EC_50_, the concentration of compound which reduces the virus titer by 50%) of binase against H1N1pdm09 was determined in MDCK-II cells. The cells were grown over night in a 48-well plate (*n* = 3) in a humidified incubator at 37 °C and 5% CO_2_. Cell monolayers were then washed and infected with H1N1pdm09 virus suspension in PBS^++^(PBS containing 1 mM MgCl_2_, and 0.9 mM CaCl_2_)/BA (PBS^++^ containing 0.21% bovine albumin and P/S) (MOI = 1) for 1 h at RT. After removing the virus inoculum, cells were then supplemented with 250 μl DMEM/BA (DMEM containing 0.21% bovine albumin and P/S) in each well containing 1 μg/ml TPCK-treated Trypsin (Sigma Aldrich, Germany) (+/−) binase at the indicated concentration and incubated for 24 h, at 37 °C and 5% CO_2_. The supernatants containing H1N1pdm09 virus were subsequently assayed for their viral titers using focus assay [24, 25].

### Hemagglutination inhibition assay (HAI)

Ligand/binase interaction: The HAI assay detects neutralizing agents to the viral hemagglutinin (HA) by measuring the inhibition of virus-mediated agglutination of erythrocytes. To determine its neutralizing ability (affinity) towards H1N1pdm09 HA receptor binding residues, binase (10^5^ U/ml) was twofold serially diluted (1:10–1:1280) in 25 μl of 1xPBS in V-shaped well plates. An equal volume (25 μl) containing four HA units of H1N1pdm09 was added and then incubated at RT for 30 min. Then 50 μl of 1% chicken erythrocytes in suspension (PBS) was added to the wells, mixed by shaking the plates on a mechanical vibrator and incubated at 4 °C until control showed dot formation.

Binase/receptor interaction: In an additional approach to analyze the ability of binase to interfere with HA binding to its receptor on the surface of the chicken erythrocytes, these were pre-incubated with binase before virus was added. Briefly, binase (10^5^ U/ml) was serially diluted in a 96-well microtiter plate in 25 μl 1xPBS and then 50 μl of 1% erythrocytes was added and incubated together for 30 min. Then, 25 μl of four hemagglutinin units (HAU) of H1N1pdm09 was then added and incubated at 4 °C for 45 min. 1% erythrocytes plus 1xPBS, binase (10^5^ U/ml) or H1N1pdm09 (4 HAU) were used as controls. Agglutination patterns were read after 45 min and the HI titer was defined as the reciprocal of the last dilution of serum that completely inhibited hemagglutination.

### Immunofluorescence assay

To detect the internalization of binase into MDCK-II cells, the cells were incubated on a glass cover slips in a 6-well plate with or without binase (10^5^ U/ml) for 4 h. Cell monolayers were then washed twice with PBS^++^ and incubated with 3.7% formaldehyde for 10 min at RT for fixation and washed. To determine the binase accumulation within the cells, cells were incubated with 0.5% Triton X-100 for 7 min at RT. After washing with PBS^++^, the cells were incubated with PBS^++^ containing 3% BSA for 20 min at RT. Following washing, rabbit polyclonal anti-binase primary antibody (1:50, provided by Dr. Vershinina, Kazan Federal University, Russia) and subsequently chicken monoclonal anti-rabbit Alexa Fluor 488 (1:1000, life technologies, USA) as secondary antibody and was added to the permeabilized cells to detect binase accumulation on- or in the cells. DAPI (0.1 mg/ml PBS/3%BA, Roth, Germany), was used for nuclei staining. The binase localization was detected using a LSM 780 confocal microscopy (Carl Zeiss, Germany). The binase accumulation on the MDCK-II cellular membrane was checked from time to time using the above-mentioned assay without 0.5% Triton X-100 treatment.

### Binase treatment

To analyze the intra-cellular effect of binase on IAV propagation, 10^5^ U/ml of binase (12 h p.i., after single-cycle virus replication) or different concentration of binase (24 h p.i., after multi-cycle virus replication) were added to confluent monolayers of MDCK-II cells in 96-well plates containing DMEM/BA medium. Cells with and without binase were pre-incubated for 4 h at 37 °C in a 5% CO_2_ atmosphere and were then further washed twice with PBS^++^ and subsequently infected with H1N1pdm09 (MOI = 1) in PBS^++^/BA for 60 min at RT. This procedure should prevent interaction between binase and virus particles outside the cells and allowed the internalization of the enzyme into the cells previous to viral infection. After removal of the virus suspension from the cell surface, the cells were further incubated for 8 h in 1 × 10^5^ U/ml binase-containing DMEM/BA medium, then the medium was removed. Cell monolayers were then washed twice with PBS^++^ and fresh binase-free DMEM/BA medium containing 1 mg/ml TPCK-treated trypsin was added. Samples of the culture media were collected at 12 h or 24 h p.i. for virus titration via focus assay [24, 25].

### Degradation analysis of vRNA within viral particles

H1N1pdm09 (200 μl) containing cell culture supernatant (1 × 10^6^ FFU/ml) was incubated with 10^5^ U/ml binase (final concentration) for 30 min at 37 °C. As a control, virus was incubated without binase. The viral RNA was then extracted and purified using “RNeasy total RNA isolation kit” (Qiagen, Germany) according to the manufacturer’s instructions. Then, vRNA of PB2 was amplified using “SuperScript III One-Step RT-PCR Platinum Taq High Fidelity” (Invitrogen, USA). Briefly, 100 ng of extracted vRNA were mixed with 25 μl “2× Reaction Mix”, 2 μl (0.4 μM) of forward and reverse universal primers [29]. The total volume was adjusted to 50 μl using nuclease-free water and then subjected to cDNA synthesis at 55 °C for 40 min, followed by pre-denaturation (94 °C for 2 min), PCR amplification (20, 25, 30, 35 cycles: 94 °C/15 s for denaturation, 58 °C/30 s for annealing and 68 °C/3 min for extension) and final extension (1 cycle: 68 °C/5 min). The RT-PCR products were detected by 1% agarose gel electrophoreses and gel documentation.

### Protein extraction and western blotting

Viral NP protein was detected via western blot analysis. Briefly, total cellular proteins from H1N1pdm09-infected MDCK-II cells (MOI = 3), either binase-treated (1 × 10^5^ U/ml, 4 h before infection and during 8 h p.i.) or left untreated, were extracted at 4, 6, 8 h p.i. as described before [30]. The protein concentration of each sample was measured using Bradford Assay Kit (Bio-Rad, USA). Cell lysates were subjected to 4–12% SDS-polyacrylamide gradient gel electrophoresis (Invitrogen, USA) and subsequent Western blotting. The samples were transferred onto polyvinylidenefluoride (PVDF) membranes (GE Healthcare, UK) according to the manufacturer’s instructions using XCell II Blot Module (Invitrogen, USA). The NP protein of H1N1pdm09 was detected using a rabbit polyclonal anti-influenza A (H1N1) virus NP protein antibody (1:1000, Thermo Scientific, USA). Beta Actin was detected as loading control using mouse monoclonal anti-beta Actin antibody (1:5000, Abcam, UK). As secondary antibodies for detection goat anti-mouse IRDye 800 CW and goat anti-rabbit IRDye 680 CW (1:15,000**,** Abcam, UK) were used and detected with Odyssey imaging systems (LI-COR, USA). Protein quantification was performed using “Quantity one” software (Bio-Rad, USA). To detect the internalized binase for binase-treated and nontreated MDCK-II cells, we performed western blot analysis as described previously.

### Primer extension

In 6-well plates, 3 wells of confluent MDCK-II cell monolayers were infected with H1N1pdm09 at a MOI of 3 either in the absence or presence of binase (1 × 10^5^ U/ml). The total RNA was extracted using Trizol reagent (Invitrogen, USA) at 8 h p.i. and processed as previously described [25, 31, 32]. The gene-specific DNA primers used were NP mRNA, cRNA (5′-ACCATTCTCCCAACAGATGC-3′) and vRNA (5′-ATGATGGAGAGTGCCAGACC-3′) specific. The cellular 5S rRNA was detected using specific primer (5′-TCCCAGGCGGTCTCCCATCC-3′) and used as an internal control. Transcription products were analyzed on 7% polyacrylamide gels containing 7 M urea in Tris-borate-EDTA (TBE) buffer and signals were detected and calculated using a molecular imager and the Quantity One software (BioRad), respectively. The increases of vRNA, mRNA and cRNA level (corrected with respect to 5S rRNA) were expressed relative to values obtained without binase treatment (arbitrarily 100%).

### RNA extraction and cDNA preparation

To characterize the effect binase on cellular mRNA MDCK-II cells were grown for 12 h in a 6-well plate and were either binase-treated (10^5^ U/ml in DMEM/BA) or left untreated (DMEM/BA) in triplicate for another 12 h. After washing with PBS^++^, cells were collected in PBS and immediately counted. 10^6^ cells from each sample were used for RNA extraction and cDNA preparation. The cells from each sample were lysed with RLT buffer (Qiagen, Germany). Total RNA was purified using an RNeasy mini Kit (Qiagen, Germany) according to manufacturer protocol. Total RNAs of each sample was eluted using the same volume of elution buffer. A concentration of 100 ng of the eluted RNA from each sample was used for reverse transcription of ATP6V0E1 and TUBA4A mRNA using MMLV RT Kit (Evrogen, Russia) and the predefined antisense primers (Section Plasmids and primers) according to manufacturer protocol.

### Real-time PCR

To quantify cellular mRNA in MDCK-II cells using qPCRmix-HS SYBR Kit (Evrogen, Russia), 4 μl of each cDNA preparation (1–100 ng of the above reaction), 0.4 μM of each (forward and reverse) primers, and 1× qPCRmix-HS SYBR (Evrogen, Russia) were mixed in a LightCycler 480 Multiwell Plate 96 (Roche Diagnostics, Switzerland) on ice in the dark and covered by LightCycler 480 Sealing Foil. Thermal cycling was done under the conditions as described above (Section Plasmids and primers). The quantification and data analysis was performed using the LightCycler 480 Service Software (Roche Diagnostics, Switzerland) and identified the binase action on cellular mRNA accumulation according to the relative fold-difference of expression levels.

### Chloramphenicol acetyl transferase (CAT) activity assay using a mini-genome system

To investigate the effect of binase on the gene expression activity of H1N1pdm09, an IAV mini-replicon system expressing a CAT reporter gene was employed. The plasmids (pHW200 for H1N1pdm09) encoding the viral polymerases (PB2, PB1, PA) and nucleoprotein (NP) were co-transfected together with pPol1-CAT-RT into 293 T cells (grown in 6 well plates) in a ratio of 1:1:1:2:2 as previously described with minor modifications [33]. Briefly, the transfection mixture consisting of 180 μl “Opti-MEM” (Thermo Fisher Scientific, USA) containing a total of 7 μg of plasmid DNA along with 15 μl “Trans-IT2020” (Mirus Bio, USA) was incubated for 45 min at RT. Then, the mixture was diluted to 1 ml using “Opti-MEM” and transferred to an 80–90% confluent 293 T cell monolayer to allow transfection. Then, the cells were incubated for 8 h at 37 °C. Afterwards, the transfection mixture was replaced with 2 ml Opti-MEM containing 0.2% BA and P/S with binase or without binase (1 × 10^4^ U/ml and 1 × 10^5^ U/ml). At 48 h post transfection, cell lysates were prepared and tested for CAT activity (1:1000 dilution) as previously described [26].

### Statistical analysis

All experiments were performed in 3–8 biological repeats. Statistical tests and graphical data presentation were performed using SigmaPlot 10.0 Software (Systat Software, Inc., USA), GraphPad Prism 5 Software (GraphPad Software, Inc., USA) and MS Excel 2010 (Microsoft Corporation, USA). All data are presented as the mean ± standard deviation of the mean (SD). The significance between two groups was determined via “Student t-test”.

### Biosafety

All experiments using infectious virus were performed in accordance with the German regulations applicable to the propagation of influenza viruses (IVs). All experiments involving pandemic influenza A (H1N1pdm09) virus were performed using biosafety level 2 (BSL2) containment laboratory approved for such use by the local authorities (RP, Giessen, Germany).

## Results

### Binase impairs H1N1pdm09 propagation in MDCK-II at non-toxic concentrations

**Fig. 1:**
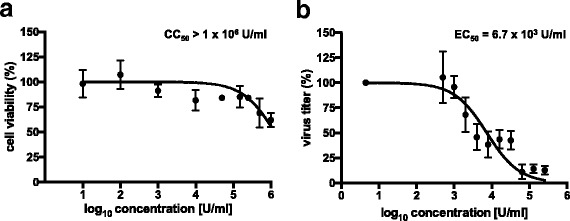
Cytotoxicity concentration 50% (CC_50_) and effective concentration 50% (EC_50_) of binase in MDCK-II cells. **a** The percentage of cell viability was determined in MDCK-II cells, incubated with different compound concentrations for 24 h, via MTT assay. **b** The percentage of virus inhibition was determined by comparing the reduction in viral titers, measured by foci assay, in H1N1pdm09-infected and binase-treated samples with mock-infected controls. Bars represent the standard deviation of mean

To elucidate the MOA of binase as anti-viral candidate against IAV, we used the MDCK-II cell line. The cytotoxic concentration of binase resulting in 50% cell death (CC_50_) was found to be higher than the highest tested binase concentration of 1000 μg/ml (approximately to 1 × 10^6^ U/ml of enzyme activity) (Fig. 1a). Next, we determined the effective concentration binase that inhibits H1N1pdm09 propagation by 50% (EC_50_) to be 6.7 × 10^3^ U/ml (Fig. 1b). Based on the CC_50_ and EC_50_ on MDCK-II cells (Fig. 1) 1 × 10^5^ U/ml were selected to be used for further experiments.

### Binase does not interfere with HA/receptor binding and does not degrade viral RNA within free virus particles

It was previously reported that pre-incubation of virus with binase resulted in decreased virus titers after infection [13, 21, 22], that binase could degrade free viral RNA [21], and that treatment of IAV-infected A549 cells led to titer reduction [22]. We therefore addressed whether binase would indeed directly interfere with the virus receptor binding activity and/or the viral RNA of the extra-cellular IAV particles.

**Fig. 2:**
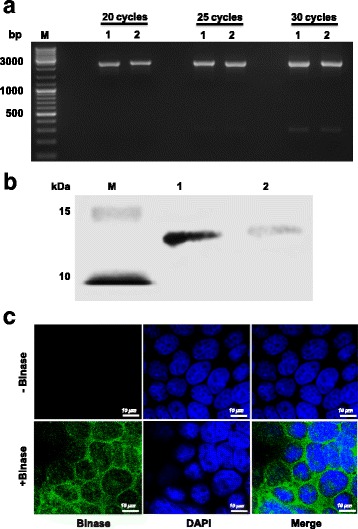
Effect of binase incubation with virus particles and MDCK-II cells. **a** To analyze the stability of genomic viral RNA (vRNA), virus particles were either left untreated (lane 1) or treated with binase (10^5^ U/ml, lane 2) and incubated for 30 min at 37 °C. RT-PCR analysis of the virus particle-protected vRNA, using specific segment primers to amplify full-length PB2 segment for different number of cycles (20, 25, 30, 35), showed that binase does not affect protected H1N1pdm09 vRNA. **b** Internalization of binase (10^5^ U/ml) into MDCK-II cells was detected by Western blot analysis 8 h post incubation (lane 2). After washing the binase-treated cells twice, total cell lysate was assayed. 100 ng of purified binase was used as a loading control (lane 1). M refers to protein size marker. **c** The internalization of binase (10^5^ U/ml) into MDCK-II cells was further analyzed by immunostaining using rabbit polyclonal anti-binase and Alexa Fluor 488-labelled (green) chicken monoclonal anti-rabbit antibodies 4 h post incubation. Cell nuclei were stained with DAPI (blue). Control cells were not incubated with binase

The HAI analysis revealed that the binase has a negligible affinity towards IAV receptor binding residues (ligand/binase interaction) or to the surface receptors on chicken erythrocytes (binase/receptor interaction) and therefore did not interfere with viral attachment to red blood cells. The HAI titers were below the limit of detection (<1:10) (Additional file 1: Figure S1). Additionally, we found that the anti-viral activity of binase was not related to a direct interaction with viral RNA within intact viral particles. Envelope- and viral ribonucleoprotein complex (vRNP)-protected viral RNA genome of H1N1pdm09 was not affected by binase following 30 min of incubation of intact viral particles with binase at the concentration of 1 × 10^5^ U/ml. As analyzed by RT-PCR at different cycle numbers (20, 25, 30 cycles), the protected full length viral RNA remained unaffected by binase, suggesting that binase did not reduce the virus titer by affecting the cell free virus particles. The results described above argue against an extra-cellular MOA of binase, but indicate that the MOA is likely to be related to an effect within the host cell (Fig. 2a).

### Binase is accumulating in the cytosol of MDCK-II cells

To investigate whether binase exerts an intra-cellular MOA, we first assessed the ability of binase to internalize into MDCK-II cells. It was demonstrated before that binase penetrated into A549 cells [23], nevertheless, we wanted to assure that this would also be the case for MDCK-II cells used in the present study. To determine the possible intra-cellular accumulation of binase in MDCK-II cells, fractions of binase-treated (1 × 10^5^ U/ml) cells were analyzed 8 h post-treatment (h p.t.) by Western blotting. The results indicated that binase could also penetrate and accumulate in MDCK-II cells (Fig. 2b**)**. Employing immunofluorescent staining and confocal microscopy, we additionally showed that binase localizes mostly in the cytosol of MDCK-II cells within 4 h p.t. (Fig. 2c). Based on these results (Fig. 1 and Fig. 2), we speculate that the intra-cellular, non-cytotoxic concentrations of binase might exert an anti-viral effect on the viral RNA genome within the infected MDCK-II cells.

### Binase impairs IAV replication by attacking viral RNA species within infected MDCK-II cells

**Fig. 3:**
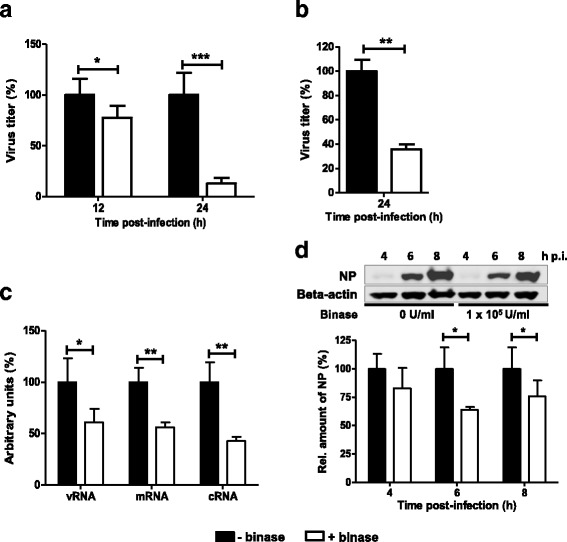
Intra-cellular effect of binase on viral replication and transcription. MDCK-II cells were pre-incubated with binase-containing media for 4 h, washed twice with PBS^++^ and infected with either H1N1pdm09 or H3N2-Vict for 1 h (MOI = 1). Inoculum was then removed and cells were washed twice with PBS^++^. DMEM/BA media, with and without binase, were added to the cell monolayers for 8 h p.i.. The cells were washed twice with PBS^++^ and fresh infection medium without binase was added to the cells. The supernatants containing progeny virions were collected 12 and 24 h p.i. for H1N1pdm09 (**a**) or 24 h p.i. for H3N2-Vict (**b**) and titrated using foci assay. **c** Effect of binase on the activity of the viral polymerase activity of H1N1pdm09 as determined by primer extension analysis. MDCK-II cells were infected (MOI = 3) with H1N1pdm09. At 8 h p.i., vRNA, mRNA, cRNA, and 5S rRNA (loading control) levels were determined. **d** Infected MDCK-II cells were treated with binase (10^5^ U/ml, white bars) for the indicated time points or left untreated (black bars) in triplicates. The effect of binase on viral NP production was analyzed by Western blotting using a NP-specific antibody and calculated by setting the percentage of untreated cells at 100%. NP-specific signals were quantified and normalized to beta-actin expression (loading control) and calculated relative to the values obtained from cells without binase treatment (arbitrarily set to 100%). Statistical analysis was performed using the Student’s t-test and two-way ANOVA, followed by Bonferroni post hoc test (* = *p* < 0.05, ** = *p* < 0.01, *** = *p* < 0.001)

Binase exerts an anti-viral effect during multicycle replication of IAV **(**Fig. 1b**)**. Based on the finding that binase can localize in the cytosol of MDCK-II at 4 h p.t. (Fig. 2b, c), we investigated the intra-cellular effect of binase on H1N1pdm09 during a single replication cycle (12 h p.i.) and multiple replication cycles (24 h p.i.). MDCK-II cells were treated with binase 4 h before infection. Cells were then washed before adding the inoculum that was subsequently replaced by binase-containing media for up to 8 h after infection. The binase-containing media was then replaced with fresh binase-free media and virus titers were determined 12 (single-cycle) and 24 h (multi-cycle) later. This way we intended to avoid any extra-cellular effect of binase on the virus particles before initial infection, as binase was absent during the infection process. Washing the cells twice after removing the binase-containing media before adding the fresh media without binase, removed all remaining extra-cellular binase from the cell monolayers. This had been verified by immunofluorescent microscopy using an anti-binase primary antibody (data not shown). By analyzing H1N1pdm09 viral titers, collected 12 h p.i., we found that binase could significantly reduce viral propagation in MDCK-II cells by 22%. However, this virus reduction effect was potentiated at 24 h p.i. to reach 87% (Fig. 3a).

To indicate that the observed binase intra-cellular anti-viral effect against H1N1pdm is not strain specific, we investigated the binase anti-viral activity against H3N2-Vict under the same conditions (binase and MDCK cells were incubated 4 h before infection and 8 h p.i., MOI for H3N2 infection = 1, supernatants were titrated 24 h p.i. for progeny virions load). Interestingly, a significant reduction in the replication of H3N2-Vict was observed (Fig. 3b).

We further determined the accumulation of vRNA, cRNA and mRNA of the NP segment in single-cycle replication (MOI = 3) by primer extension analysis using total RNA isolated at 8 h p.i. from MDCK-II-infected cells with H1N1pdm09 and either treated with 10^5^ U/ml binase or left untreated. Quantification (Fig. 3c) revealed that, compared to H1N1pdm09-infected/binase untreated MDCK-II cells, the H1N1pdm09-infected/binase treated MDCK-II cells showed significant drop in the accumulation of all viral RNA species in MDCK-II cells.

Therefore, we then checked of the NP expression using western blotting. The western blot analysis of infected and binase-treated MDCK-II cells (4 h before and after infection) analyzed 4, 6 and 8 h p.i. revealed that accumulation of the viral nucleoprotein (NP) was reduced during single-cycle replication by 36% and 24% at 6 and 8 h p.i., respectively (Fig. 3d). Taken together, these data further support our understanding that binase is intra-cellularly targeting the viral genome corresponding with a reduction of viral proteins.

### Binase does not affect expression of cellular housekeeping genes

**Fig. 4:**
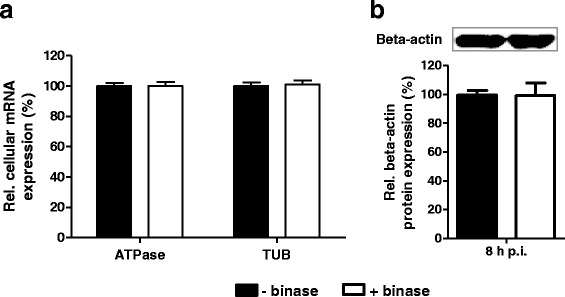
Binase is not reducing the amount of host-cell transcripts and expressed proteins. **a** Real-time RT-PCR to analyze the expression levels of the housekeeping genes mRNA transcripts ATPase (ATP6V0E1) and Tubulin (TUBA4A) in binase-treated (10^5^ U/ml) and non-treated MDCK-II cells at 12 h p.i. **b** Western blot analysis of cellular beta-actin protein expression levels in binase treated (10^5^ U/ml) and non-treated MDCK-II cells. Statistical significance was assessed using the Student’s *t*-test. *P < 0.1

To assess the specificity of binase for viral RNA, the impact of binase treatment (10^5^ U/ml) on the expression of cellular housekeeping genes in MDCK-II cells was investigated. RT-qPCR demonstrated that the transcript accumulation of the MDCK-II housekeeping genes, ATPase (*ATP6V0E1*) and Tubulin (*TUBA4A*), was not altered upon binase treatment (Fig. 4a). This was further confirmed by comparable beta-actin protein expression levels in binase-treated and untreated MDCK-II cells (Fig. 4b). Therefore, we assume that binase specifically affects viral genomic RNA and does not seem to alter cellular gene expression and translation.

### Binase reduces the gene expression of a H1N1pdm09 mini-genome system

**Fig. 5:**
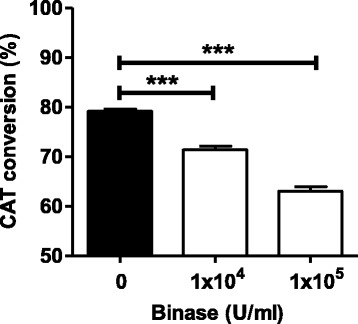
Impact of binase on the viral expression efficiency of the H1N1pdm09 RNP complex. Effect of binase on the viral expression activity of the H1N1pdm09 RNP complex was analyzed in 293 T cells transfected with plasmids expressing the polymerase subunits (PB1, PB2, and PA) and NP of H1N1pdm09 together with vRNA-like transcript (pPol1-CAT-RT) encoding the CAT reporter gene, was analyzed for CAT activity 48 h post transfection. The cells were treated with 10^4^ and 10^5^ U/ml of binase after transfection. In the control, binase was absent. Statistical significance was investigated using the Student’s *t*-test. ***P < 0.001

The viral ribonucleoprotein (vRNP) complex of IAVs is responsible for transcription and replication of the viral genomic RNA and exists as a heterotrimeric polymerase complex consisting of the PB2, PB1 and PA polymerase subunits bound to the viral RNA, which is enwrapped by the NP [34]. The plasmids encoding PB1, PB2, PA and NP genes of H1N1pdm09 and a plasmid expressing a vRNA-like Pol1 transcript encoding the open reading frame of the chloramphenicol acetyltransferase (CAT) reporter gene flanked by the 3′ and 5′ noncoding regions of the NS RNA segment of influenza A/WSN/33 (H1N1) virus (pPol1-CAT-RT) were used to reconstitute the viral RNP complex in vitro. Co-transfection of the five plasmids into 293 T cells yielded CAT protein that could be detected and quantified for its activity. The results obtained showed that the CAT activity of transfected/binase-treated cells was significantly reduced by 12% and 24% at 1 × 10^4^ U/ml and 1 × 10^5^ U/ml, respectively, in a concentration-dependent manner (Fig. 5). This observation supports the idea that internalized binase possibly affects IAV gene expression within infected mammalian cells.

## Discussion

Influenza-like illness is still a major health problem and only few anti-viral approaches are approved. These few licensed viral-protein-dependent antiviral therapies are challenged by the continuous genomic evolution of influenza A viruses (IAVs) to overcome the selective pressure due to their overuse [35]. Consequently, new viral-independent anti-viral strategies are urgently needed and represent excellent alternative anti-influenza remedies. Herein, we investigated the MOA of bacterial ribonuclease (binase), against IAV replication in MDCK-II cells in more detail to further evaluate the applicability of binase as a potential anti-influenza agent.

The current influenza anti-viral drugs target the function of the variable surface glycoproteins, NA and M2. This anti-influenza activity of NA-inhibitors and M2-blockers can be easily subverted and resisted via acquisition of distinct single or multiple amino acid (aa) residues in the corresponding viral proteins [7]. In contrast, the genome of IAV (vRNAs) has highly conserved regions, which can provide a constant site for targeting by different ribonucleases. However, some natural ribonucleases may be challenged with their high molecular weight (Mwt), existence of natural RNAase inhibitors and possible immunogenicity [36].

The bacterial ribonuclease, namely binase, is the guanyl-specific RNAase from *B. pumilus* 7P with low Mwt (12.2 kDa/109 aa residues). It is also preferred to other natural ribonuclease being non-immunogenic and resistant to mammalian RNase inhibitors in vitro and in vivo [10, 17, 20]. Binase could be used intranasally or by intravenous injection and both administrations shown an anti-viral effect of the enzyme in vivo [19, 20]. All of these features emphasize that binase has the potential to be effective anti-influenza therapy.

In this study, the results obtained from the first set experiments, including HAI and RT-PCR analysis of binase-treated virions, could exclude a detectable effect of binase on the ability of free virus particles to infect MDCK-II cells. This finding restricts the MOA of binase to an activity directly within the infected cell. Yet, previous studies [13, 21, 22] observed an anti-viral effect after pre-incubation of the virus with binase prior to infection. This discrepancy might be explained by the fact that the binase added to the virions before infection was still present during infection. Therefore, binase might have entered through the interaction of the cationic binase protein with the negative charge of the cell surface [13]. The receptors of the IAV hemagglutinin on the host cell surface carry a negative charge provided by the sialic acid [37, 38] allowing the binase to contact the cell surface via electrostatic interaction and to be internalized via endocytosis independently of the virus [39]. Since the IAV enter the cells in a similar way, binase could further utilize such endosomes for efficient uptake. Interestingly, binase seems to have the ability to penetrate into cultured mammalian cells. In addition to the MDCK-II cells used for this study, binase was previously reported to be detected in A549 cells [23].

Next we assessed the ability of binase to impair H1N1pdm09 propagation at a non-toxic anti-viral concentration, while preventing direct interaction of virus particles with binase prior to initial infection. Compared to its anti-H1N1pdm09 activity at 24 h p.i., binase showed a significant anti-viral effect at 12 h p.i., which was not very strong probably due to the restricted uptake and accumulation of the binase into the host cells during the single replication cycle. This was overcome after multicycle replication assayed after 24 h p.i.. This significant anti-viral effect was also shown for a H3N2-vict strain indicating that binase is efficiently working at 24 h p.i., regardless of the strain subtype. Based on these aforementioned observations, we advanced to test the idea of an intra-cellular MOA by investigating the effect of binase on the accumulation of viral NP, viral RNAs, mRNA of cellular housekeeping genes and on the expression activity of a mini-genome system. The quantification of viral RNAs and of viral NP in binase-treated cells, as well as of the amount of cellular mRNA encoding housekeeping genes and the expression of a reporter gene by the viral mini-genome are all showing an inhibitory effect of binase. In a similar way, it was recently shown that the cellular double-stranded-RNA-activated RNase L, a critical component of interferon-regulated anti-viral host responses, exerts its anti-viral activity during influenza virus infection by targeting specific sites in viral RNAs [40]. Unlike the cellular RNase L which has the tendency to target also the same specific sites in host cell RNAs, no significant impact on the expression of cellular housekeeping genes was observed. This has further supported the hypothesis that the IAV genome within the infected cell is the main target of binase.

In light of these findings, our study verified the favorable and specific activity of binase in inhibiting the viral replication of human IAVs in infected cells. Binase exerts its anti-viral effects by specifically degrading viral RNA species within the infected MDCK-II cells.

## Conclusion

The present study provides novel findings on the anti-viral properties and mode of action of binase. Here we show that the bacterial ribonuclease binase inhibits H1N1pdm09 propagation by degrading viral RNAs and thereby impairing the viral gene expression within infected MDCK-II cells, resulting in reduced intra-cellular viral protein accumulation. Assuming that the mode of action of binase is directed against the viral RNAs within the infected cell it should be indiscriminative and effective, regardless of the IAV subtype. This supports the idea of binase as a promising anti viral that might allow a targeted anti-influenza approach.

## Additional file

Additional file 1: Figure S1. The effect of binase on HA inhibition (HI). Twofold serially diluted binase (10^5^ U/ml) was incubated with 4HAU of H1N1pdm09 and then supplemented with 1% chicken erythrocytes (RBCs) to detect the binase/ligand interaction (binase + H1N1pdm + RBCs). Meanwhile, twofold serially diluted binase (105 U/ml) was incubated with 1% chicken erythrocytes (RBCs) and then 4 HAU of H1N1pdm09 were added to explore the binase/receptor interaction (binase + RBCs + H1N1pdm). HI assay was conducted in triplicate for each serial dilution (1:10–1:1280).1xPBS, binase (10^5^ U/ml), and H1N1pdm09 (4 HAU) were used as controls. (PDF 432 kb)

## Abbreviations

AEC: 3-amino-9-ethylcarbazole
CAT: chloramphenicol acetyltransferase
ECL: enhanced chemiluminescence
FFU: focus-forming units
h p.i.: hours post-infection
h p.t.: hours post-treatment
HA: hemagglutination
MOI: multiplicity of infection
ТРСК: L-1-Tosylamide-2-phenylethyl chloromethyl ketone
