# Supplementary figures and images for: Bacterial ribonuclease binase exerts an intra-cellular anti-viral mode of action targeting viral RNAs in influenza a virus-infected MDCK-II cells
Source: Virol J. 2018 Jan 5;15:5. doi: 10.1186/s12985-017-0915-1 (PMC5756404; doi:10.1186/s12985-017-0915-1)

Supplementary Figure (S1)

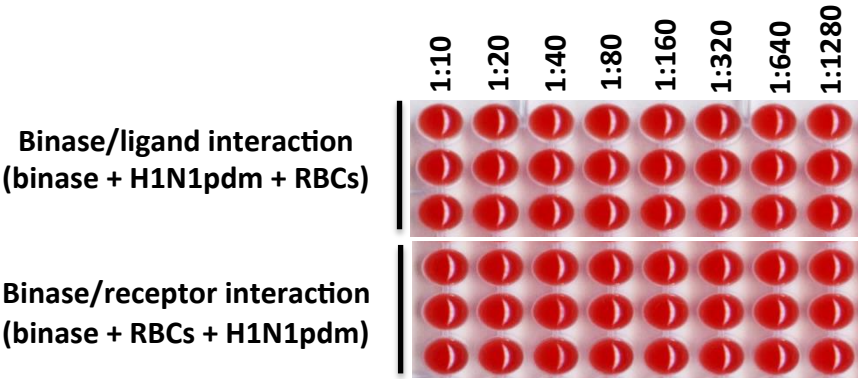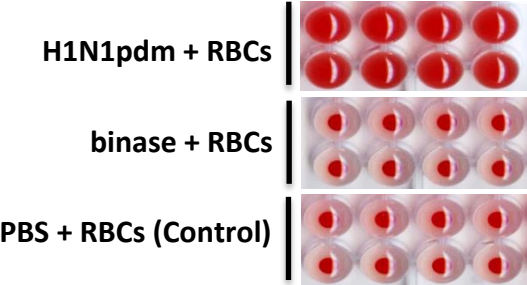

Supplement: Additional file 1: Figure S1. — The effect of binase on HA inhibition (HI). Twofold serially diluted binase (105 U/ml) was incubated with 4HAU of H1N1pdm09 and then supplemented with 1% chicken erythrocytes (RBCs) to detect the binase/ligand interaction (binase + H1N1pdm + RBCs). Meanwhile, twofold serially diluted binase (105 U/ml) was incubated with 1% chicken erythrocytes (RBCs) and then 4 HAU of H1N1pdm09 were added to explore the binase/receptor interaction (binase + RBCs + H1N1pdm). HI assay was conducted in triplicate for each serial dilution (1:10–1:1280).1xPBS, binase (105 U/ml), and H1N1pdm09 (4 HAU) were used as controls. (PDF 432 kb) [file 12985_2017_915_MOESM1_ESM.pdf]
